# Supplementary material for: What is the appropriate timing for advance care planning according to patients and their relatives? A scoping review
Source: PLoS One. 2026 Mar 20;21(3):e0345093. doi: 10.1371/journal.pone.0345093 (PMC13004342; doi:10.1371/journal.pone.0345093)
Supplement: S1 File — (DOCX) [file pone.0345093.s001.docx]

What is known about the optimal timing of starting the advance care planning process from the perspective of patients, their relatives and healthcare professionals: A scoping review

Carolien Burghout, MANP^1, 2, 3^

Sascha R. Bolt, PhD^3^

Lenny M. W. Nahar-van Venrooij, PhD^2^

Tineke J. Smilde, MD, PhD^1^

Carin C.D. van der Rijt, MD, PhD^4^

Eveline J. M. Wouters, MD, PhD^3, 5^

^1^ Department of hemato-oncology, Jeroen Bosch Hospital, ‘s-Hertogenbosch

^2^ Jeroen Bosch Academy Research, Jeroen Bosch Hospital, ‘s-Hertogenbosch

^3^ Tilburg University, School of Social and Behavioral Sciences, Department of Tranzo, Tilburg

^4^ Department of Medical Oncology, Erasmus Medical Center Cancer Institute, Rotterdam

^5^ Fontys University of Applied Science, School of Allied Health Professions, Eindhoven

**INTRODUCTION**

Advance care planning is a process that supports adults at any age or stage of health in understanding and sharing their personal values, life goals, and preferences regarding future medical care^1^. Early discussions with patients with a life-shortening illness about their preferences, may contribute to better quality of life and care.^2-4^ Speaking about end-of-life preferences and documenting them into the patient record can be helpful in proposing more appropriate treatment or end-of-life care in relation to the patients’ preferences. However, the care in the last months of life often does not meet the patients’ end-of-life preferences. As a result, patients can experience unwanted care transitions at the end of life,^5^ high symptom burden,^6^ and reduced quality of life and are not able to die at their preferred place.^5, 7, 8^ There are different factors that contribute to sub-optimal end-of-life care. Patients’ preferences are often not identified in a timely manner,^9^ marking the palliative phase is difficult ^10-12^ and healthcare professionals may find it difficult to speak about end-of life care.^13-15^ Professionals may be reluctant to speak about the end of life for many reasons, such as uncertainty about prognoses, fear of causing distress or taking away hope.^16-19^ From de perspective of the patient several barriers for initiating the process of ACP are diverse: the topic is too emotional, the perceived lack of time during the consultation,^20^ the lack of knowledge about ACP^20-21^ and uncertainty about the future.^21^

Several studies have shown that oncology patients want to talk about end-of-life care^22-24^ and expect healthcare professionals to initiate these conversations.^22, 25^ Patients with cancer stated that exploring the patients’ preferences was a precondition for constructive communication with their oncologist.^26^

Timely initiating the process of ACP and documentation of the outcomes of these discussions seem to be important, yet the optimal timing for initiating ACP to identify the patients’ preferences is not clear. There may be different perceptions about the ideal timing of initiating these conversations. Theoretically, the process of ACP can be initiated regardless of age or state of health^1^ or as soon as a patient is confronted with illness or frailty.^27^ However, previous research has shown that the process of ACP was often initiated in the last months of life,^28-32^ which might result in overtreatment during the last months of life.^2, 32, 33^ Knowing the patients’ preferences early on may lead to more conscious decision-making about whether or not to start treatment(s) at the end of life.^34^ Early initiating the process of ACP to identify the patients’ preferences enable timely application of care and treatment that are needed to meet these preferences.^35^ However, it is not clear what ‘early’ or ‘timely’ means with respect to the process of ACP to identify the patients’ preferences from the perspective of patients with a life-shortening illness and the healthcare professionals. This scoping review aims to explore what is known from the literature about the different perspectives regarding the optimal timing of starting the process of ACP to identify the patients’ preferences. Moreover, we investigate what is known about the influencing barriers, facilitators or challenges related to the timing of ACP.

**Objectives**

The research question of this scoping review is: What is known about the optimal timing of starting the advance care planning process from the perspective of patients, their relatives and healthcare professionals?

**METHODS**

*Study design*

This scoping review is conducted following the methodology for scoping reviews, ^36-37^ which includes the following six steps: 1) defining the research question, 2) identifying relevant studies, 3) study selection, 4) charting the data, 5) collating, summarizing, and reporting the results, and 6) consultation (optional). An iterative approach is taken toward searching the literature, refining the search strategy, reviewing articles for inclusion, and extracting relevant data from original research. Preferred Reporting Items for Systematic Reviews and Meta-Analyses (PRISMA) guidelines are followed using the PRISMA extension for scoping reviews (PRISMA-ScR) checklist.^38^

**Inclusion criteria**

*Type of participants/population*

This scoping review considers any manuscript that discusses the timing of the process of ACP to identify the patients’ preferences from the perspective of adult patients with the three most common life-shortening diseases: cancer, heart- and lung diseases, ^39, 40^ their relatives or their healthcare professionals. These patient groups are selected because we expect that within these groups ACP is most often initiated. Initially, we aim to explore what is known from the scientific literature. If the search results of relevant scientific articles does not reach saturation, we will conduct an additional search of grey literature.

*Concept*

Articles are eligible if they refer to the timing of starting the process of ACP to identify the preferences in adult patients with cancer, lung- or heart diseases, their relatives or their healthcare professionals.

*Context*

There are no restrictions regarding the place where care is delivered, i.e., the context within which ACP conversations take place.

*Types of sources*

Scoping reviews may include diverse types of study designs. We will consider any type of peer-reviewed publication describing a primary research study. If our initial search of the scientific literature in the databases PubMed and Cinahl does not result in saturation, we will broaden our search to the available grey literature. For this, we will consult the websites of key (national) health and palliative care organization. Grey literature may include community or policy reports, government or public agency publications, and practice guidelines. Potential websites to be searched are: The European Association for palliative care and the national websites of Zorginstituut Nederland, Palliaweb, Integraal KankerCentrum Nederland (IKNL) and Coöperatie Palliatieve Zorg Nederland (PZNL). The inclusion criteria (based on population, concept, and context) for grey literature are the same as for scientific literature. We include articles that are written in English or Dutch.

**Search strategy**

We will search PubMed and Cinahl for potential relevant scientific articles. A comprehensive search strategy was developed in conjunction with an experienced librarian at the Jeroen Bosch Hospital (JBZ) and subsequently be refined in consultation with the research

team. The strategy was validated by ensuring the retrieval of a key set of relevant studies. The search strategy was formulated using terms such as ‘advance care planning’ including relevant terms related (e.g. end-of-life conversations) to this and diverse terms for ‘timing’. These were combined with terms representing the selected life-shortening illnesses cancer, heart- and lung diseases. The following limitations or filters were applied: studies involving adults (only) and articles written in English or Dutch. Appendix 1 presents the complete search string.

**Selection of the studies**

Eligible articles must focus on the timing of ACP to identify the preferences of patients with cancer, lung- or heart diseases, their relatives or their healthcare professionals. The research team members will screen the articles independently in two phases, using the online software Covidence.^41^ First, one researcher (CB) screens all titles and abstracts. Three other researchers (EW, SB, LN) each screen a substantial part of articles on the title and abstract (phase 1). The articles are included for phase two if the title or abstract refers to the timing of ACP to identify preferences of patients with cancer, lung- or heart diseases. In phase 2, CB will screen the remaining articles for full-test eligibility. EW, SB, and LN will each screen a substantial part of full texts for eligibility. The percentage of agreement between CB and the other researchers will be calculated for both screening phases. References of the included articles will be checked for other relevant literature (snowball method). If there is any disagreement between researchers about including an article in any phase or about the information to report, they will discuss their considerations to reach consensus. If they do not come to an agreement, another researcher (TS) will be consulted.

**Extraction of the results**

One researcher (CB) screens all the included articles to extract relevant information, which is collected on a data-extraction form Three other researchers (EW, SB, LN) each screen a substantial part of included articles. The initial data extraction form contains the following general information (phase 1):

1. Author(s)

2. Year of publication

3. Country of origin

4. Publication type (journal article or grey literature)

5. Study design (quantitative, qualitative, or mixed)

6. Care setting

7. Study population and number of participants (if present)

When the grey literature needs to be consulted, additional information is charted, including the name of the issuing organization and the type of document.

The research question focuses on the optimal timing of starting the process of ACP, including related barriers, facilitators and challenges from the perspective of patients, their relatives or healthcare professional. Therefore, we will also extract the following information (phase 2):

8. Results about optimal timing of ACP to identify the preferences from the

perspective of patients, their relatives or their healthcare professionals

9. Relevant barriers, facilitators or challenges related to the timing of ACP, as identified by the patient, their relatives or healthcare professional.

After extracting results (i.e., relevant fragments of text from the included articles) as meant in phase 2, these will be coded using thematic analysis, as described by Braun and Clarke.^42^ The thematic analysis can be approached in six phases:

1. Familiarizing with the data – reading and familiarizing with the relevant text fragments extracted from the included studies

2. Generating initial codes – labeling meaningful segments of these texts through open coding. The results of the optimal timing of the process of ACP will be coded deductive (e.g. regardless of age or state of health, when confronted with illness of frailty or at the last months of life). The results of the described barriers, facilitators and challenges will be coded in an inductive manner.

3. Searching for themes – clustering initial codes to form overarching themes

4. Reviewing potential themes – discussing the initial themes within the research team

5. Defining and naming themes – reaching consensus within the research team about the appropriate formulation of themes and what they entail

6. Producing the product/overarching themes – reporting and/or depicting the final themes supported by quoted fragments from the original articles

Analyses will be performed with ATLAS.ti. Due to the exploratory nature of this scoping review, the data extraction template may be adapted as the data extraction proceeds.

**Presentation of the results**

We will present the results of the search and screening process in a PRISMA flowchart. Next, we will provide an overview/table of the general information extracted from our included studies. We will summarize and discuss the optimal timing of the process of ACP to identify the patients’ preferences and various described barriers, facilitators or challenges from both the perspective of patients, their relatives or professionals by means of the identified themes. Lastly, we will describe the remaining gaps in the literature, as well as the implications for practice and future research.

**Declaration of Conflicting Interests**

The author(s) declared no potential conflicts of interest with respect to

the research, authorship, and/or publication of this article.

**Funding**

The author(s) received no financial support for the research, authorship,

and/or publication of this article.

**References**

1. Sudore R, Lum HD, You JJ. et al. Defining Advance Care Planning for Adults: A Consensus Definition From a Multidisciplinary Delphi Panel. J Pain Symptom manage.2017;53(5):821-832. [doi:10.1016/j.jpainsymman.2016.12.331](https://pubmed.ncbi.nlm.nih.gov/28062339/)
2. Burghout C, Nahar-van Venrooij LMW, Bolt SR, Smilde TJ., Wouters EJM. Benefits of structured advance care plan in end-of-life care planning among older oncology

patients: A retrospective pilot study. J of Pall Care.2023;38(1):30-40. [doi:10.1177/08258597221119660](https://journals.sagepub.com/doi/abs/10.1177/08258597221119660)

1. Narsavage GL, Chen Y-J, Korn B, Elk R. The potential of palliative care for patients with respiratory diseases. Breathe (Sheff).2017;13(4):278-289.

[doi:10.1183/20734735.014217](https://pubmed.ncbi.nlm.nih.gov/29209422/)

1. Schichtel M, We, B, Perera R., Onakpoya I. The effect of advance care planning on heart failure: a systematic review and meta-analysis. J. Gen Intern Med.2020;35(3): 874-884. [doi: 10.1007/s11606-019-05482-w](https://pubmed.ncbi.nlm.nih.gov/31720968/)
2. Abarshi E, Echteveld M, van den Block L, Donker G., Deliens L, Onwuteaka-Philipsen B. Transitions between care settings and the end of life in the Netherlands: result from a nationwide study. Palliat Med.2010:166-74. [doi: 10.1177/0269216309351381](DOI:%2010.1177/0269216309351381)
3. Merchant SJ, Brogly SB, Booth CM, Goldie C, Nanji S, Patel SV et al. Palliative care and symptom burden in the last year of life: a population-based study of patients with gastrointestinal cancer. Ann Surg Oncol.2019: 2336-2345. [doi: 10.1245/s10434-019-07320-z.](https://pubmed.ncbi.nlm.nih.gov/30969388/)
4. Bell CL, Somogyi-Zalud E, Masaki KH. Factors associated with congruence between preferred and actual place of death. J Pain Symptom Manage.2010:591-604. [doi: 10.1016/j.jpainsymman.2009.07.007](https://pubmed.ncbi.nlm.nih.gov/20116205/)
5. Tang ST & Mccorkle R. Determinants of congruence between the preferred and actual place of death for terminally ill cancer patients. J Pallia Care.2003:230-237. PMID: 14959592
6. Glaudemans JJ, Moll van Charante EP, Willems DL. Advance care planning in primary care, only for severely ill patients? A structured review. Fam Pract.2015;32(1):16-26. [doi: 10.1093/fampra/cmu074](https://pubmed.ncbi.nlm.nih.gov/25381010/)
7. Epiphaniou E, Shipman C, Harding R. et al. Coordination of end-of-life care

for patients with lung cancer and those with advanced COPD: are there transferable lessons? A longitudinal qualitative study. Prim Care Respir J.2014; 23(1):46-51.

[doi: 10.4104/pcrj.2014.00004](https://pubmed.ncbi.nlm.nih.gov/24477771/)

1. Low J, Pattenden J, Candy B, Beattie JM, Jones L. Palliative care in advanced heart failure: an international review of the perspectives of recipients and health care professionals on care provision. J Card Fail.2011;17(3):231-52. [doi:10.1016/j.cardfail.2010.10.003](https://pubmed.ncbi.nlm.nih.gov/21362532/)
2. Murray SA, Boyd K, Sheikh A. Palliative care in chronic illness. BMJ.2005;330(7492):611-12. [doi: 10.1136/bmj.330.7492.611](https://pubmed.ncbi.nlm.nih.gov/15774965/)
3. Ethier JL, Paramsothy T, You JJ, Fowler R, Gandhi S. Perceived barriers to goals of care discussions with patients with advanced cancer and their families in the ambulatory setting: A multicenter survey of oncologists. J Palliat Care.2018;33(3):125-142. [doi: 10.1177/0825859718762287](https://pubmed.ncbi.nlm.nih.gov/29607704/)
4. Periyakoil VS, Neri E, Kraemer H. No easy talk: a mixed methods study of doctor reported barriers to conducting effective end-of-life conversations with diverse patients. PLoS One 2015; 10:e0122321. [doi: 10.1371/journal.pone.0122321](https://pubmed.ncbi.nlm.nih.gov/25902309/)
5. Piggott KL, Patel A, Wong A. et al. Breaking silence: A survey of barriers to goals of care discussions from the perspective of oncology practitioners. BMC Cancer.2019;19(1):130. [doi: 10.1186/s12885-019-5333-x](https://pubmed.ncbi.nlm.nih.gov/30736754/)
6. Brighton LJ, & Bristowe K. Communication in palliative care: talking about the end of life, before the end of life. Postgrad Med J.2016; 92(1090):466-470. [doi:10.1136/postgradmedj-2015-133368](https://pubmed.ncbi.nlm.nih.gov/27153866/)
7. Blackwood DH, Walker D, Mythen MG, Taylor RM, Vindrola-Padros C. Barriers to advance care planning with patients as perceived by nurses and other healthcare professionals: a systematic review. J Clinic Nurs.2019;28(23-24):4276-4297. [doi: 10.1111/jocn.15049](https://pubmed.ncbi.nlm.nih.gov/31494997/)
8. Owusuaa C, van Lent LGG, van t Spijker A, van der Rijt CCD, van der Heide A. Discussing prognosis and the end of life with patients with advanced cancer or COPD: a qualitative study. PLoS One.2022:e0274201. [doi: 10.1371/journal.pone.0274201](https://pubmed.ncbi.nlm.nih.gov/36084060/)
9. Treaplethon D, Chung RY, Wong YS, Wong ELY, Kiang N, Chau PYK, Woo J, Chung VCH, Yeoh EK. Care Toward the End of Life in Older Populations and Its Implementation Facilitators and Barriers: A Scoping Review. J Am Med Dir Assoc.2018;18(12):1000-1009. [doi.org/10.1016/j.jamda.2017.04.010](https://pubmed.ncbi.nlm.nih.gov/28623155/)
10. Bernard C, tan A, Slaven M, et al. Exploring patient-reported barriers to advance care planning in family practice. BMC family practice 2020; 21:94. [doi: 10.1186/s12875-020-01167-0](https://pubmed.ncbi.nlm.nih.gov/32450812/)
11. Peck V, Valiani S, Tanuseputro P, et al. Advance care planning after hospital discharge: qualitative analyses of facilitators and barriers from patient interviews. BMC palliat Care.2018;17(1):127. [doi: 10.1186/s12904-018-0379-0](https://pubmed.ncbi.nlm.nih.gov/30518345/)
12. Kubi B, Istl AC, Lee KT, Conca-Cheng A, Johnston FM. Advance care planning in cancer: patient preferences for personnel and timing. JCO Oncol Pract.2020;16(9):e-875-e883. [doi: 10.1200/JOP.19.00367](https://pubmed.ncbi.nlm.nih.gov/32282265/)
13. Waller A, Turon H, Bryant J, Zucca A, Evans TJ, Sanson-Fisher R. Medical oncology outpatients’ preferences and experiences with advanced care planning: A cross-sectional study. BMC Cancer.2019;19(1):63. [doi: 10.1186/s12885-019-5272-6](https://pubmed.ncbi.nlm.nih.gov/30642289/)
14. Zwakman M, Jabbarian LJ, Van Delden JJM. et al. Advance care planning: A systematic review about experiences of patients with a life-threatening or life-limiting illness. Palliat Med.2018;32(8):1305-1321. [doi: 10.1177/0269216318784474](https://pubmed.ncbi.nlm.nih.gov/29956558/)
15. Davison SN. Facilitating advance care planning for patients with end-stage renal disease: the patient perspective. Clin J Am Soc Nephrol. 2006;1(5):1023-1028. [doi: 10.2215/CJN.01050306](https://pubmed.ncbi.nlm.nih.gov/17699322/)
16. Westendorp J, Evers AWM, Stouthard JML et al. Mind your words: Oncologists' communication that potentially harms patients with advanced cancer: A survey on patient perspectives. Cancer. 2022; 128(5):1133-1140. [doi: 10.1002/cncr.34018](https://pubmed.ncbi.nlm.nih.gov/34762305/)
17. Raad voor Volksgezondheid & Samenleving. Leven met het einde in zicht. De waarde van een brede benadering*.* 2022. [Online](https://www.raadrvs.nl/actueel/nieuws/2022/03/09/leven-met-het-einde-in-zicht)
18. Boddaert MS, Pereira C, Adema J. et al. Inappropriate end-of-life cancer care in a generalist and specialist palliative care model: a nationwide retrospective population-based observational study. BMJ Support Palliat Care.2020;0:1-9. [doi:10.1136/bmjspcare-2020-002302](https://pubmed.ncbi.nlm.nih.gov/33355176/)
19. Hui D, Hannon BL, Zimmerman C, Bruera E. Improving patient and caregiver outcomes in oncology: teambased, timely and targeted palliative care. CA Cancer J Clin.2018;68(5):356-376. [PMC6179926](http://www.ncbi.nlm.nih.gov/pmc/articles/pmc6179926/)
20. Gidwani R, Joyce N, Kinosian B. et al. Gap between recommendations and practice of palliative care and hospice in cancer patients. J Palliat Med.2016;19(9):957-963. [doi: 10.1089/jpm.2015.0514](https://pubmed.ncbi.nlm.nih.gov/27228478/)
21. Van der Padt-Pruijsten A, Leys MBL, Oomen-de Hoop E, Van der Heide A, Van der Rijt CCD. Effects of implementation of a standardized Palliative Care Pathway for patients with advanced cancer in a hospital: a prospective pre- and post-intervention study. J pain symptom manage.2021;62(3):451-459. [doi: 10.1016/j.jpainsymman.2021.02.003](https://pubmed.ncbi.nlm.nih.gov/33561492/)
22. Vektis. Factsheet palliatieve zorg. 2021. Available at: https://www.vektis.nl. Accessed 12 June 2022.
23. Koekoek B. Regie over de plaats van sterven. Een kwantitatieve en kwalitatieve verkenning*.* 2014. [Online](https://www.vptz.nl/onderzoek-publicaties/regie-plaats-sterven-kwantitatieve-en-kwalitatieve-verkenning/#:~:text=Er%20blijkt%20een%20discrepantie%20te,in%20het%20gewenste%20hospice%20overleden.)
24. Hoerger M, Greer JA, Jackson VA. et al. Defining the elements of early palliative care that are associated with patient-documented outcomes and the delivery of end-of-life care. J Clin Oncol.2018;36(11):1096-1102. [doi:10.1200/JCO.2017.75.6676](https://pubmed.ncbi.nlm.nih.gov/29474102/)
25. Bernacki RE, & Block SD. Communication about serious illness care goals. A review and syntheses of best practices. JAMA intern Med.2014;174(12):1994-2003. [doi: 10.1001/jamainternmed.2014.5271](https://pubmed.ncbi.nlm.nih.gov/25330167/)
26. Arksey H, O'Malley L. Scoping studies: towards a methodological framework. International Journal of Social Research Methodology. 2005;8(1):19
27. Levac D, Colquhoun H, O'Brien KK. Scoping studies: advancing the methodology. Implementation science: IS. 2010;5:69
28. Tricco AC, Lillie E, Zarin W, O'Brien KK, Colquhoun H, Levac D, et al. PRISMA Extension for Scoping Reviews (PRISMA-ScR): Checklist and Explanation. Annals of Internal Medicine. 2018;169(7):467. [doi.org/10.7326/M18-0850](https://www.acpjournals.org/doi/10.7326/M18-0850)
29. Centers for disease control and prevention. Available at: <https://wwwcdc.gov>. Accessed 12 June 2022.
30. European chronic disease alliance. Available at: <https://alliancechronicdiseases.org/>. Accessed 12 June 2022.
31. COVIDENCE systematic review software, Veritas health innovation, Melbourne, Australia. Available at <https://www.covidence.org/>. Accessed 15 September 2022.
32. Braun V, & Clarke V. (2006). Using thematic analysis in psychology. Qualitative Research in Psychology;3:77–101.

**Appendix 1 Searchstring**

**Pubmed**

((((((("Advance Care Planning"[Mesh] OR "ACP"[tiab]) OR (("proactive care"[tiab] OR "advance care"[tiab] OR "end of life"[tiab] OR "palliative"[tiab]) AND ("communication*"[tiab] OR "conversation*"[tiab] OR "discuss*"[tiab] OR "dialogue*"[tiab] OR "discourse*"[tiab] OR "talk*"[tiab] OR "consultation"[tiab] OR "prefer*"[tiab] OR "wish*"[tiab] OR "plan*"[tiab] OR "care"[tiab])))) **AND** ("time*"[tiab] OR "timing"[tiab] OR "initiat*"[tiab] OR "early"[tiab] OR "start*"[tiab])) **AND** ((("Neoplasms"[Mesh]) OR (("Heart Diseases"[Mesh] OR "Lung Diseases"[Mesh]) AND ("chronic*"[tiab] OR "advanced"[tiab] OR "palliative"[tiab]))) OR (("tumor*"[tiab] OR "tumour*"[tiab] OR "cancer*"[tiab] OR "malignan*"[tiab] OR "carcinoma*"[tiab] OR "hematolo*"[tiab] OR "haematolo*"[tiab] OR "fibros*"[tiab]) AND ("chronic*"[tiab] OR "advanced"[tiab] OR "palliative"[tiab])) OR ((("heart"[tiab] OR "cardiac"[tiab] OR "lung"[tiab] OR "pulmonar*"[tiab]) AND ("illn*"[tiab] OR "diseas*"[tiab] OR "disorder"[tiab] OR "condition*"[tiab])) AND ("chronic*"[tiab] OR "advanced"[tiab] OR "palliative"[tiab]))))) **NOT** ("Adolescent"[Mesh] OR "Child"[Mesh] OR "Infant"[Mesh] OR "adolescen*"[tiab] OR "child*"[tiab] OR "schoolchild*"[tiab] OR "infant*"[tiab] OR "girl*"[tiab] OR "boy"[tiab] "boys"[tiab] OR "teen"[tiab] OR "teens"[tiab] OR "teenager*"[tiab] OR "youth*"[tiab] OR "pediatr*["tiab] OR "paediatr*"[tiab] OR "puber*"[tiab])) **NOT** ("Adult"[Mesh] OR "adult*"[tiab] OR "man"[tiab] OR "men"[tiab] OR "woman"[tiab] OR "women"[tiab])

**Cinahl**

(MH "Advance Care Planning" OR TI “ACP” OR AB “ACP”) OR ((TI (“proactive” OR “advance care” OR “end of life” OR “palliative”) OR AB (“proactive” OR “advance care” OR “end of life” OR “palliative”)) AND (TI (“communication*” OR “conversation*” OR “discuss*” OR “dialogue*” OR “discourse*” OR “talk*” OR “consultation” OR “prefer*” OR “wish*” OR “plan*” OR “care”) OR AB (“communication*” OR “conversation*” OR “discuss*” OR “dialogue*” OR “discourse*” OR “talk*” OR “consultation” OR “prefer*” OR “wish*” OR “plan*” OR “care”))) **AND** (TI (“time*” OR “timing” OR “initiat*” OR “early” OR “start*”) OR AB (“time*” OR “timing” OR “initiat*” OR “early” OR “start*”)) **AND** (MH "Neoplasms" OR ((MH ("Heart Diseases" OR "Lung Diseases")) AND (TI (“chronic*” OR “advanced” OR “palliative”) OR AB (“chronic*” OR “advanced” OR “palliative”)))) OR ((TI (“Tumor*” OR “Cancer*” OR “Malignan*” OR “Carcinoma*” OR “Hematolo*” OR “haematolo*” OR “Fibros*”) OR AB (“Tumor*” OR “Cancer*” OR “Malignan*” OR “Carcinoma*” OR “Hematolo*” OR “haematolo*” OR “Fibros*”)) AND (TI (“chronic*” OR “advanced” OR “palliative”) OR AB (“chronic*” OR “advanced” OR “palliative”))) OR ((TI (“Heart” OR “Cardiac” OR “Lung” OR “Pulmonar*”) OR AB (“Heart” OR “Cardiac” OR “Lung” OR “Pulmonar*”)) AND (TI (“illn*” OR “diseas*” OR “disorder” OR “condition*”) OR AB (“illn*” OR “diseas*”OR “disorder” OR “condition*”)) AND (TI (“chronic*” OR “advanced” OR “palliative”) OR AB (“chronic*” OR “advanced” OR “palliative”))) **NOT** ((MH ("Adolescence" OR "Child+") OR TI (“adolescen*” OR “child*” OR “schoolchild*” OR “infant*” OR “girl*” OR “boy” OR “boys” OR “teen” OR “teens” OR “teenager*” OR “youth*” OR “pediatr*” OR “paediatr*” OR “puber*”) OR AB (“adolescen*” OR “child*” OR “schoolchild*” OR “infant*” OR “girl*” OR “boy” OR “boys” OR “teen” OR “teens” OR “teenager*” OR “youth*” OR “pediatr*” OR “paediatr*” OR “puber*”)) **NOT** (MH ("Adult+") OR TI (“adult*” OR “man” OR “men” OR “woman” OR “women”) OR AB (“adult*” OR “man” OR “men” OR “woman” OR “women”)))
