# Supplementary material for: What is the appropriate timing for advance care planning according to patients and their relatives? A scoping review
Source: PLoS One. 2026 Mar 20;21(3):e0345093. doi: 10.1371/journal.pone.0345093 (PMC13004342; doi:10.1371/journal.pone.0345093)
Supplement: S3 File — (DOCX) [file pone.0345093.s003.docx]

**S3 Search string**

**PubMed**

((("Advance Care Planning"[MeSH] OR "ACP"[Tiab] OR (("proactive care"[Tiab] OR "advance care"[Tiab] OR "end of life"[Tiab] OR "palliative"[Tiab]) AND ("communication*"[Tiab] OR "conversation*"[Tiab] OR "discuss*"[Tiab] OR "dialogue*"[Tiab] OR "discourse*"[Tiab] OR "talk*"[Tiab] OR "consultation"[Tiab] OR "prefer*"[Tiab] OR "wish*"[Tiab] OR "plan*"[Tiab] OR "care"[Tiab]))) AND ("time*"[Tiab] OR "timing"[Tiab] OR "initiat*"[Tiab] OR "early"[Tiab] OR "start*"[Tiab]) AND ("Neoplasms"[MeSH] OR (("Heart Diseases"[MeSH] OR "Lung Diseases"[MeSH]) AND ("chronic*"[Tiab] OR "advanced"[Tiab] OR "palliative"[Tiab])) OR (("tumor*"[Tiab] OR "tumour*"[Tiab] OR "cancer*"[Tiab] OR "malignan*"[Tiab] OR "carcinoma*"[Tiab] OR "hematolo*"[Tiab] OR "haematolo*"[Tiab] OR "fibros*"[Tiab]) AND ("chronic*"[Tiab] OR "advanced"[Tiab] OR "palliative"[Tiab])) OR (("heart"[Tiab] OR "cardiac"[Tiab] OR "lung"[Tiab] OR "pulmonar*"[Tiab]) AND ("illn*"[Tiab] OR "diseas*"[Tiab] OR "disorder"[Tiab] OR "condition*"[Tiab]) AND ("chronic*"[Tiab] OR "advanced"[Tiab] OR "palliative"[Tiab])))) NOT (((("Adolescent"[MeSH] OR "Child"[MeSH] OR "Infant"[MeSH] OR "adolescen*"[Tiab] OR "child*"[Tiab] OR "schoolchild*"[Tiab] OR "infant*"[Tiab] OR "girl*"[Tiab] OR "boy"[Tiab]) AND "boys"[Tiab]) OR "teen"[Tiab] OR "teens"[Tiab] OR "teenager*"[Tiab] OR "youth*"[Tiab] OR "pediatr*"[Tiab] OR "paediatr*"[Tiab] OR "puber*"[Tiab]) NOT ("Adult"[MeSH] OR "adult*"[Tiab] OR "man"[Tiab] OR "men"[Tiab] OR "woman"[Tiab] OR "women"[Tiab])))

**CINAHL**

(((MH "Advance Care Planning" OR TI “ACP” OR AB “ACP”) OR ((TI (“proactive” OR “advance care” OR “end of life” OR “palliative”) OR AB (“proactive” OR “advance care” OR “end of life” OR “palliative”)) AND (TI (“communication*” OR “conversation*” OR “discuss*” OR “dialogue*” OR “discourse*” OR “talk*” OR “consultation” OR “prefer*” OR “wish*” OR “plan*” OR “care”) OR AB (“communication*” OR “conversation*” OR “discuss*” OR “dialogue*” OR “discourse*” OR “talk*” OR “consultation” OR “prefer*” OR “wish*” OR “plan*” OR “care”)))) and ((TI (“time*” OR “timing” OR “initiat*” OR “early” OR “start*”) OR AB (“time*” OR “timing” OR “initiat*” OR “early” OR “start*”))) and (((MH "Neoplasms" OR ((MH ("Heart Diseases" OR "Lung Diseases")) AND (TI (“chronic*” OR “advanced” OR “palliative”) OR AB (“chronic*” OR “advanced” OR “palliative”))) OR ((TI (“Tumor*” OR “Cancer*” OR “Malignan*” OR “Carcinoma*” OR “Hematolo*” OR “haematolo*” OR “Fibros*”) OR AB (“Tumor*” OR “Cancer*” OR “Malignan*” OR “Carcinoma*” OR “Hematolo*” OR “haematolo*” OR “Fibros*”)) AND (TI (“chronic*” OR “advanced” OR “palliative”) OR AB (“chronic*” OR “advanced” OR “palliative”))) OR ((TI (“Heart” OR “Cardiac” OR “Lung” OR “Pulmonar*”) OR AB (“Heart” OR “Cardiac” OR “Lung” OR “Pulmonar*”)) AND (TI (“illn*” OR “diseas*” OR “disorder” OR “condition*”) OR AB (“illn*” OR “diseas*”OR “disorder” OR “condition*”)) AND (TI (“chronic*” OR “advanced” OR “palliative”) OR AB (“chronic*” OR “advanced” OR “palliative”)))))) NOT ((MH ("Adolescence" OR "Child+") OR TI (“adolescen*” OR “child*” OR “schoolchild*” OR “infant*” OR “girl*” OR “boy” OR “boys” OR “teen” OR “teens” OR “teenager*” OR “youth*” OR “pediatr*” OR “paediatr*” OR “puber*”) OR AB (“adolescen*” OR “child*” OR “schoolchild*” OR “infant*” OR “girl*” OR “boy” OR “boys” OR “teen” OR “teens” OR “teenager*” OR “youth*” OR “pediatr*” OR “paediatr*” OR “puber*”)) NOT (MH ("Adult+") OR TI (“adult*” OR “man” OR “men” OR “woman” OR “women”) OR AB (“adult*” OR “man” OR “men” OR “woman” OR “women”)))
