# Supplementary material for: What is the appropriate timing for advance care planning according to patients and their relatives? A scoping review
Source: PLoS One. 2026 Mar 20;21(3):e0345093. doi: 10.1371/journal.pone.0345093 (PMC13004342; doi:10.1371/journal.pone.0345093)
Supplement: S4 File — (DOCX) [file pone.0345093.s004.docx]

**S4 Code tree**

| **Subtheme** | **Codes** | |
| --- | --- | --- |
|  | **Perspective of patients** | **Perspective of relatives** |
| During healthy state | *Appropriate*  In a healthy state (before illness/cancer) (Ca) |  |
| Early/earlier in disease process | *Appropriate*  After diagnosis/early in the disease process (Ca/LD)  Before a patient becomes ill (Ca/HD/LD)  As soon as possible (Ca/LD)  ACP should happen earlier (often started too late) in disease trajectory (Ca/LD) | *Appropriate*  After diagnosis/early in the disease process (Ca/LD)  Before a patient becomes ill (Ca)  ACP should happen earlier (often started too late) (LD) |
|  | *Inappropriate*  ACP is too early (I am not in that stage yet) (Ca/LD)  Around diagnose is too early (Ca)  No early ACP when emotionally charge, mentally exhausted or impaired cognition (HD)  No ACP while there is hope to get better (losing hope) (Ca)  No ACP when the diagnosis is still doubtful (Ca)  Early ACP may cause harm (Ca)  Early ACP may suggest that end-of-life is approaching (LD) | *Inappropriate*  Early ACP may feel like giving up (Ca) |
| Before deterioration | *Appropriate*  When patients are well/not in a crisis situation (Ca/HD/LD)  Before cognitive impairment (Ca/HD) | *Appropriate*  When patients are well/not in a crisis situation (Ca/HD)  Before cognitive impairment (Ca) |
| After deterioration | *Appropriate*  After deterioration (Ca/LD) |  |
| Before treatment | *Appropriate*  When starting treatment/discussions about treatment (Ca)  Before starting treatment (Ca)  Before LVAD placement (HD)  Prior to MCS implant (HD) | *Appropriate*  When starting treatment/discussions about treatment (Ca)  Before LVAD placement (HD) |
| During treatment | *Appropriate*  During treatment course (Ca) | *Appropriate*  During treatment course (Ca) |
| After treatment | *Appropriate*  At the end of therapy (Ca)  After LVAD placement (HD) | *Appropriate*  At the end of therapy (Ca) |
|  | *Inappropriate*  Not after LVAD placement (HD)  Not after surgery (HD) |  |
| End-stage (palliative/terminal phase) | *Appropriate*  After advanced diagnosis/incurable disease (Ca)  At the end stage of disease (Ca/LD)  During transition to palliative care (Ca)  In a terminal phase/disease (LD) | *Appropriate*  In a terminal phase/disease (Ca)  When treatment is no longer meaningful (Ca)  Three months before death (Ca) |
|  | *Inappropriate*  Not when feeling well in the palliative phase (Ca) |  |

Note: Ca: cancer; LD: lung disease; HD: heart disease; ACP: advance care planning; EoL: end of Life; COPD: chronic obstructive pulmonary disease; LVAD: left ventricular assist device; MCS: mechanical circulatory support

| **Theme: Facilitators related to the appropriate timing of ACP** | | |
| --- | --- | --- |
| **Subtheme** | **Codes** | |
|  | **Perspective of patients** | **Perspective of relatives** |
| Clear information about prognose and expectations | Prognostic information (Ca/LD)  Information about what to expect (Ca/LD)  Timing depends on the topic (Ca) | Information about what to expect (LD)  Timing depend on the topic (Ca) |
| Acceptance/readiness | Close relationship with professionals (Ca)  After patient-initiated discussions (Ca)  When patients are ready (Ca/HD)  Patients need a ‘warning shot’ to prepare for the discussion (Ca/LD)  After assessing their own physical condition (LD)  Not when the patient is not in the right mind (HD) |  |
| ACP as part of standard care | At annual check-up (Ca)  At annual COPD review (LD)  ACP should be initiated naturally/in support service (Ca)  Initiated ACP may stimulate follow-up discussions (Ca) |  |
| Clarity about who initiates ACP | Professionals are in the lead to start ACP conversations (Ca)  Professionals must regularly access each patient with respect to prognose and emotional state (Ca) |  |

Note: Ca: cancer; LD: lung disease; HD: heart disease; ACP: advance care planning; EoL: end of Life; COPD: chronic obstructive pulmonary disease

| **Theme: Challenges related to the appropriate timing of ACP** | | |
| --- | --- | --- |
| **Subtheme** | **Codes** | |
|  | **Perspective of patients** | **Perspective of relatives** |
| Patient-related | | |
| Individual needs | ACP depends on the patient/individual needs/individual-based (Ca/LD/HD)  Is someone ever ready for ACP? (Ca)  Readiness to talk is individual (Ca)  Patient must accept mortality before initiate ACP (LD)  I want to discuss ACP with my family first (Ca) | ACP depends on the patient/individual needs/individual-based (Ca/HD) |
| Coping | I take one day at the time (no need to talk about ACP) (Ca)  Keeping positive (not engaging ACP) as a protective coping measure (Ca)  Keeping positive (focus on cure) means not participating in ACP (Ca)  Laziness, distractibility and fatalism are reasons for not engaging ACP (Ca)  Postponing to a more appropriate time (Ca)  ACP indicates a negative outcome (Ca)  ACP indicates the seriousness of the illness (Ca)  Tension between avoiding and facing EoL (Ca/HD)  Talking about ACP/death is difficult/uncomfortable (Ca/LD)  ACP only when absolutely necessary (LD) | Keeping positive (not engaging ACP) as a protective coping measure (Ca) |
| Mutual emotional protection | Hesitancy about ACP discussions with family (Ca)  If I choose one person to carry out my wishes, it will upset others (Ca)  Perspective of timing is different between patients and surrogates (HD) | Initiating ACP is challenging for family members (Ca)  Not want to hurt each other (Ca)  Perspective of timing is different between patients and surrogates (HD) |
| Illness- or treatment- related | | |
| Prognosis or illness uncertainty | Illness uncertainty may trigger or delay ACP (Ca)  Perceived good health and prognosis may delay ACP (Ca) | Unpredictable disease trajectory (in LVAD) hinders ACP (HD) |
| Professional-related | | |
| Reluctance | Professionals reluctance to initiate (Ca) |  |
| Time constraints | Lacking a time that was right (Ca)  Professionals lack of time (Ca) | Lacking a time that was right (Ca) |

Note: Ca: cancer; LD: lung disease; HD: heart disease; ACP: advance care planning; EoL: end of Life; COPD: chronic obstructive pulmonary disease

LVAD: left ventricular assist device

| **Theme: Triggers related to the appropriate timing of ACP** | | |
| --- | --- | --- |
| **Subtheme** | **Codes** | |
|  | **Perspective of patients** | **Perspective of relatives** |
| Patient-related | | |
| Age  Experiences in life | At a certain age (Ca) |  |
|  | After physical and mental experiences (HD)  After witnessing others dealing with EoL issues (LD) |  |
| Trigger points in illness | | |
| Turning points | After ‘turning points’ such as: infection triggering change, a new metastasis, increasing pain, increasing dyspnea, loss of a function, decline in their general condition and stopping chemotherapy (Ca/LD)  After recurrence of disease/treatment failure/poor prognosis (Ca/LD)  After occurring symptoms/complications (Ca/HD/LD) | In case of potential medical problems (Ca) |

Note: Ca: cancer; LD: lung disease; HD: heart disease; ACP: advance care planning; EoL: end of Life; COPD: chronic obstructive pulmonary disease
