# Supplementary material for: What is the appropriate timing for advance care planning according to patients and their relatives? A scoping review
Source: PLoS One. 2026 Mar 20;21(3):e0345093. doi: 10.1371/journal.pone.0345093 (PMC13004342; doi:10.1371/journal.pone.0345093)
Supplement: Attachment — Submitted filename: Response to reviewers.docx [file pone.0345093.s006.docx]

**Point by point response to the reviewers**

**Reviewer 1**

Comment: Study Selection: Could you please clarify whether the screening method was conducted in accordance with established guidelines? After reviewing both the full text and the protocol, it was not entirely clear whether double screening was applied to all documents during the screening process. For example:

- Protocol: “In phase 2, CB will screen the remaining articles for full-text eligibility. EW, SB, and LN will each screen a substantial part of full texts for eligibility.”
- Manuscript: “CB screened 8867 records for eligibility based on title and abstract, with 854 (9.6%) also independently screened by EW, LN, or SB. This led to 84 articles selected for full-text review. CB assessed all full texts, with 15 (17.9%) additionally reviewed independently by EW, LN, or SB, resulting in 25 articles meeting the inclusion criteria. The updated search up to May 2025 retrieved 2816 additional records, including 518 duplicates. CB screened the remaining 2298 records, selecting 22 for full-text review, of which four met the inclusion criteria.”

If the screening process adhered to the guidelines, please make this explicit in the manuscript to avoid any ambiguity.

Response: *We have revised the study selection paragraph in the manuscript to clarify the screening procedure and that it was in accordance with established guidelines and the study protocol. The text now describes the selection process more clearly, ensuring transparency and methodological rigor.*

Revisions:

- Method section, page 5, line 94-101: The online software Covidence ([www.covidence.org](http://www.covidence.org)) [29] was used **to support** for the screening and selection process of papers **according to international guidelines [28, 30]. Initially, titles and abstracts were screened to exclude articles that were irrelevant or beyond the scope of this review conform the study protocol (see S1 protocol).** CB screened all titles and abstracts, while EW, LN, and SB independently screened a subset until consensus was reached. **After the initial screening phase, articles considered suitable for full-text review were further assessed.** CB screened all articles in the full-text phase, and EW, LN, or SB independently screened subsets. In both phases, discrepancies were resolved through discussion. Reasons for the exclusion of full-text papers were reported.
- Result section, page 6, line 128-140: The initial search in PubMed and CINAHL in April 2023 retrieved 9,448 records, of which 581 were duplicates. The remaining 8,867 records were screened for eligibility based on title and abstract by CB, with a subset of 854 (9.6%) independently double-screened by EW, LN, or SB. **Following the double-screening phase, discrepancies were resolved, after which the remaining title and abstract screening was completed by CB.** This resulted in 84 articles selected for full-text review. CB assessed all full texts, with a subset of 15 (17.9%) independently **double-**reviewed by EW, LN, or SB, resulting in 25 articles meeting the inclusion criteria. The updated search up to May 2025 retrieved 2,816 additional records, including 518 duplicates. CB screened **all** the remaining 2,298 records, selecting 22 for full-text review, of which four met the inclusion criteria. **Any uncertainties regarding study inclusion or exclusion were resolved through consultation with LN until consensus was achieved.** Together, both searches yielded 29 papers that were fit for inclusion. Fig 1 presents the PRISMA flow chart of the study selection process.

Comment: Table 3 – “Uncertainty” subtheme. Regarding the subtheme “Uncertainty” under professional-related challenges related to the timing of ACP: even after reviewing the code tree, the rationale for setting this subtheme was unclear. It is not mentioned in the Results section, although the Discussion addresses it in detail. Providing a more detailed description of the results related to this subtheme would enhance the clarity and persuasiveness of your findings.

Response: *Upon reflection, we agreed that the subtheme “Uncertainty” did not fully capture the patients’ experiences. We have therefore renamed the subtheme to “Reluctance” to better reflect the findings.*

Revisions:

- Abstract: page 2, line 35: Timing challenges were categorized as patient- (individual needs, coping, mutual protection), illness- (prognosis or illness related uncertainty) and professional-related (**reluctance,** time constraints)
- Table 3: page18: we replaced ‘uncertainty’ with ‘reluctance’
- Result section, page 19, line 214-217: Diverse challenges related to the timing of ACP were mentioned by patients and relatives, and these included the subthemes patient-related (individual needs, coping, mutual protection), illness/treatment-related (prognosis or illness uncertainty), and professional-related challenges (**reluctance,** time constraints) (Table 3 and S4 Code tree).
- Result section, page 19, line 234: **Lastly, patients described professional-related challenges related to reluctance with regard to the initiation of ACP. Patients reported that ACP conversations were often postponed or avoided because professionals appeared reluctant to introduce discussions about future health deterioration or end-of-life care [54]. In addition, patients and relatives experienced that time taken to engage ACP conversations was insufficient [53,54].**

Comment: S1 Search String Please use “PubMed” consistently in the main text.

Response: *We have reviewed the manuscript and ensured consistent use of “PubMed” throughout the text. Additionally, to further clarify the databases searched, we slightly revised the results section to explicitly mention both PubMed and CINAHL.*

Revision:

- Result section, page 6, line 128: The initial search in **PubMed and CINAHL** in April 2023 retrieved 9,448 records, of which 581 were duplicates.

Comment: Figure 1: The notation “n=11.165” and “n=11.059” uses a period (.) instead of a comma (,).

A comma would be preferable for consistency. Additionally, this notation appears only in these items, creating inconsistency. Standardizing the format would improve readability.

Response: *We have aligned the notation of participant numbers in both Figure 1 and the main text, replacing periods with commas.*

Revisions:

- Figure 1: all periods replaced with commas
- Result section, page 6, line 128-140: The initial search in PubMed and CINAHL in April 2023 retrieved **9,448** records, of which 581 were duplicates. The remaining **8,867** records were screened for eligibility based on title and abstract by CB, with a subset of 854 (9.6%) independently double-screened by EW, LN, or SB. Following the double-screening phase, discrepancies were resolved, after which the remaining title and abstract screening was completed by CB. This resulted in 84 articles selected for full-text review. CB assessed all full texts, with a subset of 15 (17.9%) independently double-reviewed by EW, LN, or SB, resulting in 25 articles meeting the inclusion criteria.

The updated search up to May 2025 retrieved **2,816** additional records, including 518 duplicates. CB screened all the remaining **2,298** records, selecting 22 for full-text review, of which four met the inclusion criteria. Any uncertainties regarding study inclusion or exclusion were resolved through consultation with LN until consensus was achieved. Together, both searches yielded 29 papers that were fit for inclusion. Fig 1 presents the PRISMA flow chart of the study selection process.

Comment: Table 1: Consider organizing the order (e.g., by year) to make the research overview easier to grasp.

Response: *To enhance clarity and readability, we reorganized Table 1 by disease category, as recommended by the other reviewer, making it easier to see the number of studies per group. Within each disease category, studies are now grouped by perspective (patients, relatives, or both) and listed chronologically from most recent to oldest. This revised structure addresses the recommendations of both reviewers.*

Revision:

- See Table 1, page 8 (see also at the end of this letter)

Comment: Table 2 & S2 (Code Tree): Including citation symbols to indicate the source literature for each data point would be helpful. Currently, it is difficult to identify which literature the data in Table 2 originates from. Adding these references would also strengthen the credibility of the data.

Response: *We have added references to Tables 2 and 3. This revision provide a clearer and more complete overview of the evidence.*

Revisions:

- See Table 2, page 15 (see also page 8 of this letter)
- See Table 3, page 17 (see also page 8 of this letter)

**Revised tables**

**Table 1 Overview of the included studies**

| Author | Year of publication | Country of origin | Aim of the study | Study design | Type of data collection | Setting | Participants | Number of  participants |
| --- | --- | --- | --- | --- | --- | --- | --- | --- |
| Articles Oncology | | | | | | | | |
| Perspective of patients | | | | | | | | |
| Pendleton D. | 2025 | USA | To characterize patient beliefs and experiences pertaining to ACP discussions, and to explore factors that may be associated with patient experiences. | Quantitative research | Survey | Hospital | Patients with cancer | 100 |
| Trevizan F.B. | 2023 | Brazil | To identify the patients who are most likely to participate in discussions about palliative care (PC) and advance care planning (ACP), and to determine their preferred timing and approach of discussion. | Mixed method | Survey, interview | Hospital | Patients with cancer | 115 |
| Zhu T. | 2023 | The Netherlands, Belgium, Slovenia, Italy, Denmark, and the United Kingdom | To explore the experiences of patients with advanced cancer regarding the timing of ACP. | Quantitative research | Survey | Hospital | Patients with cancer | 288 |
| Kubi B. | 2020 | USA | To determine oncology patients’ preferences surrounding ACP with a focus on the choice of which health care providers to have the conversation with and the timing of conversations. | Mixed method | Survey, other (medical record review) | Hospital | Patients with cancer | 200 |
| Seifart C. | 2020 | Germany | To investigate gender differences concerning the content, the desired time point, and the mode of initiation of EOL conversations in cancer patients. | Qualitative research | Interview | Mixed setting (hospital, rehabilitation clinic) | Patients with cancer | 186 |
| Fliedner M. | 2019 | Switzerland | To explore advanced cancer patients’ experiences with a structured early palliative care intervention, its acceptability and impact on the patients’ life including influencing factors. | Qualitative research | Interview | Hospital | Patients with cancer | 20 |
| Ólafsdóttir K.L. | 2018 | Iceland | To explore the experiences of patients newly diagnosed with advanced lung cancer and their family members of engaging in a person-centered and structured ACP discussion. | Qualitative research | Interview | Hospital | Patients with cancer | 7 |
| Sherman A.C. | 2018 | USA | To deepen our understanding of preparedness for EOL care, from the perspective of patients themselves. | Qualitative research | Interview | Hospital | Patients with cancer | 13 |
| Michael N. | 2013 | Australia | To further an appreciation of how cancer patients consider ACP so that patients’ responses could inform the development of an ACP program in an Australian Cancer Centre. | Mixed method | Interview, other (Vignettes) | Hospital | Patients with cancer | 18 |
| Barnes K.A. | 2011 | United Kingdom | To explore the suitability, nature, and efficacy of ACP discussions from the perspectives of oncology patients and carers. | Qualitative research | Other (audiotaped consultations) | Mixed setting (hospital, hospice) | Patients with cancer | 40 |
| Perspective of relatives | | | | | | | | |
| Hayashi Y. | 2022 | Japan | To determine the (1) status of end-of-life discussions in general wards, (2) associated factors, and (3) impact of end-of-life discussions on outcomes. | Quantitative research | Survey | Hospital | Relatives of patients with cancer | 119 |
| Perspective of patients and relatives | | | | | | | | |
| Sripaew S. | 2024 | Thailand | To explore the perceptions of older cancer patients and their families towards ACP engagement | Qualitative research | Interview | Hospital | Patients with cancer, and relatives | 32 (16 patients, 16 relatives) |
| Canny A. | 2022 | Scotland | To assess the feasibility and acceptability to patients, carers, and GPs of a primary care ACP intervention. | Mixed method | Interview, survey | Mixed setting (general practice, hospital) | Patients with cancer and relatives | 23 patients, unknown amount relatives |
| Ikander T. | 2022 | Denmark | To investigate current nursing practice related to end-of-life discussions with incurable lung cancer patients and their family caregivers from the perspectives of patients, family caregivers, and nurses in an oncology outpatient clinic. | Mixed method | Focus group, interview, observation | Hospital | Patients with cancer and relatives | 28 (9 patients, 8 relatives) |
| Sato A. | 2022 | Japan | To assess the need for a question prompt list (QPL) that encourages end-of-life discussions between patients with advanced cancer and their physicians. | Qualitative research | Interview, focus group | Hospital | Patients with cancer, and relatives | 18 (5 patients, 7 relatives) |
| Rodi H. | 2021 | Australia | To provide more clarity regarding ACP processes in Australian oncology settings to enable widespread uptake across the cancer system. | Quantitative research | Survey | Other (national online study) | Patients with cancer and relatives | 705 (440 patients, 265 relatives) |
| Cutshall N.R. | 2020 | USA | To review a social media tweet chat to understand barriers to ACP experienced by brain tumor stakeholders. | Qualitative research | Other (tweet analyses) | Other (Forum on internet) | Patients with cancer and relatives | 52 tweet chat participants (21 patients, 5 caregivers) |
| Fritz L. | 2020 | The Netherlands | To develop an ACP program specifically for glioblastoma patients and assess the preferred content, the best time to introduce such a program in the disease trajectory, and possible barriers and facilitators for participation and implementation. | Qualitative research | Interview, focus group | Hospital | Patients with cancer and relatives | 23 (8 patient-proxy dyads, 5 proxies of deceased patients) |
| Toguri J.T. | 2020 | Canada | To: 1) explore patients’ and families’ understanding, experience and reflections on ACP, as well as what they need from their physicians during the process; 2) explore physicians’ views of ACP, including their experiences with initiating ACP and views on ACP training. | Qualitative research | Interview | Hospital | Patients with cancer, relatives | 18 (4 patients, 4 relatives) |
| Booker R. | 2018 | Canada | To understand the barriers to and facilitators of ACP in hematopoietic stem cell transplantation. | Qualitative research | Interview | Hospital | Patients with cancer and relatives | 13 (6 patients, 5 relatives) |
| Barnes K. | 2007 | United Kingdom | To explore the acceptability of an interview schedule, designed to encourage conversations regarding future care; and to explore the suitability of such discussions and inquire about their possible timing, nature and impact. | Qualitative research | Focus group | Mixed setting (hospital, hospice) | Patients with cancer and relatives | 22 (18 patients, 4 relatives) |
| Articles lung diseases | | | | | | | | |
| Perspective of patients | | | | | | | | |
| Tavares N. | 2020 | United Kingdom | To understand the preferences of patients with chronic obstructive pulmonary disease for discussions about palliative and advance care planning with clinicians. | Qualitative research | Interview | Mixed setting (primary care, respiratory outpatient clinics, research department) | Patients with lung disease | 33 |
| Nguyen M. | 2013 | Canada | To explore the perceived ACP needs of people with COPD at different illness severities and how these are met by a DVD discussing ACP. | Qualitative research | Interview | Hospital | Patients with lung disease | 12 |
| Perspective of relatives | | | | | | | | |
| Pooler C. | 2018 | Canada | To explore bereaved caregivers’ experiences of IPF patients’ end-of-life care with the palliative approach initiated at the first visit to the clinic. | Qualitative research | Interview | Hospital | Relatives in lung disease | 8 |
| Perspective of patients and relatives | | | | | | | | |
| Kalluri M. | 2022 | Canada | To explore perspectives of IPF patients, family caregivers, and healthcare professionals on ACP-related experiences to understand and inform an ACP framework to guide clinicians and facilitate early, meaningful conversations. | Qualitative research | Interview | Hospital | Patients with lung-disease and relatives | 20 (5 patients, 5 relatives) |
| Articles heart diseases | | | | | | | | |
| Perspective of patients | | | | | | | | |
| Dzou T. | 2022 | USA | To identify and describe MCS (mechanical circulatory support) individuals’ perceptions of opportunities and challenges for ongoing ACP communication. | Qualitative research | Interview | Hospital | Patients with heart disease | 24 |
| Perspective of relatives | | | | | | | | |
| Chuzi S. | 2021 | USA | To understand bereaved caregiver and clinician perceptions of end-of-life discussions and care for patients with DT LVADs. | Qualitative research | Interview | Hospital | Relatives of patients with heart disease | 7 |
| Perspective of patients and relatives | | | | | | | | |
| Metzger M. | 2016 | USA | To increase our understanding of patients’ and surrogates’ experience of engaging in ACP discussions, specifically how and why these discussions may benefit patients with LVADs and their families. | Qualitative research | Interview | Hospital | Patients with heart diseases and relatives | 28 (14 patients/surrogate pairs) |
| Articles with a mixed patient population | | | | | | | | |
| Hjorth N.E. | 2018 | Norway | To explore pulmonary patients’ needs and preferences regarding ACP in order to prepare for the introduction of ACP in Norwegian hospitals | Qualitative research | Focus group | Hospital | Patients with cancer or lung disease | 13 |

**Table 2 Appropriateness of ACP timing according to patients and relatives**

| **Subtheme** | **Timing** | **Stated by** | | | | | | **References** | **Illustrating quotes or text passages** | **Type*** |
| --- | --- | --- | --- | --- | --- | --- | --- | --- | --- | --- |
|  |  | **Patient** | | | **Relative** | | |  |  |  |
|  |  | **Ca** | **HD** | **LD** | **Ca** | **HD** | **LD** |  |  |  |
| During healthy state | Appropriate | ✓ |  |  |  |  |  | **43****,** **45****,** **51** | “(..) with one individual expressing that ACP should be discussed prior to any serious diagnosis” [42]. | Patient’s quote |
|  |  |  |  |  |  |  |  |  |  |  |
| Early/earlier in disease process | Appropriate | ✓ | ✓ | ✓ | ✓ |  | ✓ | **34****-****46****,** **59** | "To me, I think that is something that (ACP) should be at the beginning” [37]. | Patient’s quote |
|  | **Inappropriate** | **✓** | **✓** | **✓** | **✓** |  |  | **22****,** **35****,** **48-50****,** **59** |  |  |
| Before deterioration | Appropriate | ✓ | ✓ | ✓ | ✓ | ✓ |  | **37****,** **39****,** **45****,** **46****,** **48****,** **50****,** **52****,** **53** | “Well, anytime (now). Whenever it’s convenient with anyone like . . . You know? Because at the moment I’m in good health. So, I would really like to, I suppose, talk to someone” [47]. | Patient’s quote |
| After deterioration | Appropriate | ✓ |  | ✓ |  |  |  | **48****,** **51****,** **54** | “If...Dr [x] said to me, “look...it’s flaring up again.” and if it was, then I think I’d say, “well, now let’s plan” [53]. | Patient’s quote |
| Before treatment | Appropriate | ✓ | ✓ |  | ✓ | ✓ |  | **45****,** **50****,** **52****,** **55****,** **56** | “For patients who preferred to have discussions during the course of their cancer therapy (6.5%), they typically preferred to have them before starting therapy” [44]. | Result (author) |
| During treatment | Appropriate | ✓ |  |  | ✓ |  |  | **44****,** **55** | “Most patients and family caregivers wanted to talk about the future at different times throughout treatment” [43]. | Result (author) |
| After treatment | Appropriate | ✓ | ✓ |  | ✓ |  |  | **36****,** **52****,** **56** | “In total, the majority of the interviewed cancer patients would like to talk about any topic when their disease is getting worse (58%); 27.5% prefer to talk at the end of therapy” [55]. | Result (author) |
|  | **Inappropriate** |  | **✓** |  |  |  |  | **50****,** **52** |  |  |
| End stage (palliative/terminal phase) | Appropriate | ✓ |  | ✓ | ✓ |  |  | **36****,** **37****,** **41****,** **55****,** **57** | “Whereas some wanted to discuss their EoL as soon as possible, others wanted to delay it until the very end” [56]. | Result (author) |
|  | Inappropriate | ✓ |  |  |  |  |  | **44** |  |  |

Note: ACP: advance care planning, Ca: cancer; HD: heart disease; LD: lung disease; EoL: end-of-life

**Table 3	Facilitators, challenges and triggers related to the appropriate timing of ACP according to patients and relatives**

| **Facilitators related to the appropriate timing of ACP** | | | | | | | | | |
| --- | --- | --- | --- | --- | --- | --- | --- | --- | --- |
| **Subtheme** | **Stated by** | | | | | | **Illustrating quotes or text passages** | **Type*** | **References** |
|  | **Patient** | | | **Relative** | | |  |  |  |
|  | **Ca** | **HD** | **LD** | **Ca** | **HD** | **LD** |  |  |  |
| Having clear information about prognosis and expectations | ✓ |  | ✓ | ✓ |  | ✓ | “I want to know what to expect, what’s coming down the line. To me, I think that is something that should be at the beginning” [37]. | Patient’s quote | **38****,** **43** |
| Acceptance/ readiness | ✓ | ✓ | ✓ |  |  |  | “You need to fit it in with them when they are ready” [51]. | Patient’s quote | **35****,** **37****,** **41,** **44****,** **48****,** **52****,** **54** |
| ACP as part of standard care | ✓ |  | ✓ |  |  |  | “Participant comments revealed a wish to incorporate ACP discussions into the annual physical checkup” [44]. | Result (author) | **39****,** **45****,** **48** |
| Clarity about who initiates ACP | ✓ |  |  |  |  |  | “A small number of patients said they would take the lead from health professionals on when to have conversations” [53]. | Result (author) | **47****,** **51****,** **54** |
| **Challenges related to the appropriate timing of ACP** | | | | | | | | | |
| Patient-related | | | | | | | | | |
| Individual needs | ✓ | ✓ | ✓ | ✓ | ✓ |  | “The participants varied widely in their opinions of the best time to broach ACP. Whereas some wanted to discuss their EoL as soon as possible, others wanted to delay it until the very end. Overall, it seems that most of the participants simply requested the medical team be sensitive to their needs on an individual basis” [57]. | Result (author) | **22****,** **34-37,** **39****,** **47****,** **48****,** **52****,** **54****,** **58****,** **59** |
| Coping | ✓ | ✓ | ✓ | ✓ |  |  | “Several patients explained that they preferred to concentrate on positive outcomes and felt that ACP was an indication or concession that treatment would be unsuccessful: ‘I think that’s [ACP] an acceptance that it is going to go bad” [59]. | Result (author)/ patient’s quote | **22****,** **39****,** **44****,** **47****,** **48****,** **54** **59****,** **60** |
| Mutual emotional protection | ✓ | ✓ |  | ✓ | ✓ |  | “Because the subject of death and dying was uncomfortable, the participants seemed hesitant about initiating the discussion with their family, to protect both them and their family. One patient wondered if people were ever ready to discuss these matters: “Well, are you ever ready? [silence] I hope that I will live a little bit longer, hopefully” [38]. | Result (author)/ patient’s quote | **39****,** **44****,** **47****,** **52** |
| Illness/ treatment-related | | | | | | | | | |
| Prognosis or illness uncertainty | ✓ |  |  |  | ✓ |  | “I could see him deteriorating. His energy level was deteriorating. But did I suspect that he was doing to die? No. So was I prepared? No.” [60]. | Relative’s quote | **59****,** **61** |
| Professional-related | | | | | | | | | |
| **Reluctance** | ✓ |  |  |  |  |  | “Over a third said their doctors were reluctant to introduce such topics. They always try to be positive.... upbeat... So, he’s not going...to say, “What happens if it goes wrong?” He doesn’t want to discuss it” [53]. | Result (author)/ patient’s quote | **54** |
| Time constraints | ✓ |  |  | ✓ |  |  | “The doctors...are very busy...so I have not talked to them, because it is probably quite a lengthy subject” [53]. | Patient’s quote | **53****,** **54** |
| **Triggers related to the appropriate timing of ACP** | | | | | | | | | |
| Patient-related | | | | | | | | | |
| Age | ✓ |  |  |  |  |  | “Many patients commented on the possible benefit of establishing guidelines for beginning ACP discussions by a certain age, similar to other screening interventions” [44]. | Result (author) | **45** |
| Experiences in life |  | ✓ | ✓ |  |  |  | “When I was hospitalized here, there was a man next to me. He may have passed away by now, but let’s just say that wasn’t the way that I would like to end my life. I plan on taking every precaution necessary to never end up that way” [57]. | Patient’s quote | **50****,** **58** |
| Trigger points in illness | | | | | | | | | |
| Turning points | ✓ | ✓ | ✓ | ✓ |  |  | “The participants named key turning points that often were consistent with major medical changes. Examples of such turning points were an infection triggering change, a new metastasis, increasing pain, increasing dyspnea, loss of a function, decline in their general condition and stopping chemotherapy” [36]. | Result (author) | **22****,** **37****,** **43****,** **48****,** **50****,** **51****,** **56** |

Note: ACP: advance care planning, Ca: cancer; HD: heart disease; LD: lung disease; EoL: end-of-life

* Some excerpts are direct quotes from patients or relatives, while others are author interpretations

**Reviewer 2:**

Comment: A scoping review is a method for comprehensively mapping existing literature, but this study focused only on two databases: PubMed and CINAHL. While I understand that experienced librarians were involved, I would like to ask you to explain why these two search engines were selected.

Response: *We selected PubMed and CINAHL for their comprehensive coverage of primary studies in medicine, nursing, and allied health relevant to ACP for patients with cancer, lung, or heart diseases. Other databases were considered, but given their different focus and scope, we did not expect them to yield many additional relevant studies, making PubMed and CINAHL the most focused and manageable choice. We have clarified in the manuscript that PubMed and CINAHL were selected for their comprehensive coverage of primary studies in medicine, nursing, and allied health, relevant to ACP for patients with cancer, lung, or heart diseases.*

Revision:

- Method section, page 4, line 70: **The literature search was conducted in two databases (PubMed and CINAHL) selected for their comprehensive coverage of original studies in the fields of medicine, nursing, and allied health relevant to ACP for patients with cancer, lung, or heart diseases.**

Comment: Is it correct to assume that all articles were independently screened using "CB" and "EW, LN, SB"?

Response: *To clarify, CB screened all records for eligibility, while EW, LN, and SB independently screened a subset of records at each stage, until consensus was reached. CB independently continued screening and discussed any uncertainties with the coauthors. We have revised the study selection paragraph to make this process more explicit, including which subsets were independently double-screened, in accordance with the study protocol and established guidelines.*

Revisions:

- Method section, page 5, line 94-101: The online software Covidence (www.covidence.org) [29] was used **to support** for the screening and selection process of papers **according to international guidelines [28, 30].** **Initially, titles and abstracts were screened to exclude articles that were irrelevant or beyond the scope of this review conform the study protocol (see S1 protocol).** CB screened all titles and abstracts, while EW, LN, and SB independently screened a subset until consensus was reached. **After the initial screening phase, articles considered suitable for full-text review were further assessed**. CB screened all articles in the full-text phase, **and** EW, LN, or SB independently screened subsets. In both phases, discrepancies were resolved through discussion. Reasons for the exclusion of full-text papers were reported.
- Result section, page 6, line 128-140: The initial search in **PubMed and CINAHL** in April 2023 retrieved 9,448 records, of which 581 were duplicates. The remaining 8,867 records were screened for eligibility based on title and abstract by CB, with a subset of 854 (9.6%) independently double-screened by EW, LN, or SB. **Following the double-screening phase, discrepancies were resolved, after which the remaining title and abstract screening was completed by CB.** This resulted in 84 articles selected for full-text review. CB assessed all full texts, **with a subset** of 15 (17.9%) **independently double-**reviewed by EW, LN, or SB, resulting in 25 articles meeting the inclusion criteria. The updated search up to May 2025 retrieved 2,816 additional records, including 518 duplicates. CB screened **all** the remaining 2,298 records, selecting 22 for full-text review, of which four met the inclusion criteria. **Any uncertainties regarding study inclusion or exclusion were resolved through consultation with LN until consensus was achieved.** Together, both searches yielded 29 papers that were fit for inclusion. Fig 1 presents the PRISMA flow chart of the study selection process.

Comment: It is expected that results will differ depending on the target disease (cancer, heart disease, lung disease). Please clarify why you chose not to address differences by disease.

Response: *While differences in ACP timing and perspectives between disease groups are plausible, our review focused on mapping the different perspectives of patients and relatives in ACP qualitatively, rather than comparing findings between disease groups. In addition, most included studies were conducted in oncology and the perspectives of patients with heart or lung disease, as well as their relatives, were underrepresented. Thus, formal comparisons between disease groups were not considered feasible. We have now explicitly clarified this in the manuscript.*

Revision:

- Limitation, page 22, line 317: Most studies were conducted in the field of oncology, with only a few including patients or relatives with lung or heart disease**. In addition, the perspective of relatives was generally underrepresented. Although it is plausible that the optimal timing of ACP may differ across conditions such as cancer, heart disease, or lung disease, the qualitative nature of the included results and the predominance of studies conducted in oncology, together with the underrepresentation of studies including relatives, did not allow for meaningful comparisons between disease groups or between patients and relatives. Rather than focusing on potential differences, this review aimed to synthesize the perspectives of patients and relatives across life-shortening illnesses in order to identify overarching patterns. Despite these limitations, the results across these various perspectives support the notion that the optimal timing for initiating ACP is highly individual among all of these groups.**

Comment: As the authors state, this study integrates studies from widely different cultural backgrounds, healthcare systems, and definitions of ACP, so caution is required when generalizing themes. In particular, patients' and families' perceptions of "appropriate timing" may differ significantly between countries with well-established ACP systems (e.g., the Netherlands, the UK) and those without (e.g., Brazil, Asian countries). Therefore, please consider further how national, cultural, and institutional differences may affect the results.

Response: *We have expanded the text to discuss how cultural, national, and institutional factors may influence perceptions of ACP timing, highlighting differences across Western vs. non-Western contexts and between countries with well-established ACP systems and those without.*

Revision:

- Discussion, page 20, line 254: The wide variation in the perceived appropriate timing of ACP conversations about end-of-life suggests that ACP timing is a highly individual matter. This underlines previous notions of the importance of understanding each patient’s personal circumstances [62, 63] and social background [64] when healthcare professionals want to initiate ACP conversations. **Furthermore, cultural, national, and institutional factors may shape perceptions of the appropriate timing of ACP. Most included studies were conducted in Western countries, where patient autonomy is highly valued [65], which may facilitate openness to early ACP conversations. In contrast, in non-Western or family-centered cultures, decision-making may be more collective or deferential to medical authority, influencing both willingness to engage in ACP and perceptions of timing [66]. Similarly, countries with well-established ACP systems and training for care providers may support earlier and more structured discussions, whereas in countries without such frameworks ACP may occur later or structured in a less formal manner [67].** **Individual patient characteristics, social background, as well as cultural, national, and institutional contexts are likely to contribute to the wide variation in perceived timing observed in this review, highlighting the importance of considering these multiple contexts when initiating ACP conversations.**

Comment: A strength of this study is that it addressed the perspectives of both patients and families, but the results do not clearly show the differences between the two. Consider indicating the differences between the patient and family perspectives.

Response: *While differences in ACP timing between patients and relatives are plausible, our review focused on mapping the different perspectives of patients and relatives in ACP, rather than to compare findings between the diverse perspectives. We have now clarified this point in the manuscript.*

Revision:

- Limitation, page 22, line 317: Most studies were conducted in the field of oncology, with only a few including patients or relatives with lung or heart disease**. In addition, the perspective of relatives was generally underrepresented. Although it is plausible that the optimal timing of ACP may differ across conditions such as cancer, heart disease, or lung disease, the qualitative nature of the included results and the predominance of studies conducted in oncology, together with the underrepresentation of studies including relatives, did not allow for meaningful comparisons between disease groups or between patients and relatives. Rather than focusing on potential differences, this review aimed to synthesize the perspectives of patients and relatives across life-shortening illnesses in order to identify overarching patterns. Despite these limitations, the results across these various perspectives support the notion that the optimal timing for initiating ACP is highly individual among all of these groups.**

Comment: The detailed structure of Table 1 is acceptable, but it would be easier to read if it were classified by disease.

Response: *To enhance clarity and readability, we reorganized Table 1 by disease category, as recommended, making it easier to see the number of studies per group. Within each disease category, studies are now grouped by perspective (patients, relatives, or both) and listed chronologically from most recent to oldest, as requested by the other reviewer. This revised structure addresses the recommendations of both reviewers.*

Revision:

- See Table 1, page 8 (see also page 8 of this letter)

Comment: Because ACP was handled differently in each study, it would be desirable to provide a brief summary at the beginning

Response: *We have added a brief summary at the beginning of the manuscript clarifying that ACP involves structured communication to explore patients’ values, preferences, and goals. Although operationalized differently across studies, ACP supports shared decision-making and alignment of care with patients’ preferences.*

Revisions:

- Introduction, page 3, line 41-46: **Advance care planning (ACP) generally refers to a process of structured communication in which patients explore and articulate their values, preferences, and goals for future medical care [1, 2]. Although ACP is operationalized differently across studies, it typically involves high-quality conversations that help patients reflect on what matters most to them when facing treatment decisions. Ideally, ACP involves collaboration between clinicians, patients, and their loved ones to consider how care should be approached if, or when the patient’s health deteriorates**
