# Supplementary material for: What is the appropriate timing for advance care planning according to patients and their relatives? A scoping review
Source: PLoS One. 2026 Mar 20;21(3):e0345093. doi: 10.1371/journal.pone.0345093 (PMC13004342; doi:10.1371/journal.pone.0345093)
Supplement: Attachment — Submitted filename: Point by point response to the reviewers.docx [file pone.0345093.s007.docx]

**Point by point response to the reviewers**

**Reviewer 1**

Comment: Thank you for clarifying the study selection procedure and for adding detailed descriptions in both the Methods and Results sections. The transparency of the screening process has clearly improved. Since you now state that the procedure was conducted “according to international guidelines,” I would encourage you to specify which guideline or methodological source you are referring to. If your approach is based on resource-efficient screening methods—such as those described by Garritty et al. (2022)—it would be helpful to cite this work directly and briefly explain that partial double-screening is an accepted method within this framework. Without this clarification, readers may assume adherence to more traditional guidelines (e.g., PRISMA or JBI), which generally recommend independent screening by two reviewers for all records. Therefore, explicitly naming and citing the guideline or methodological paper that supports your approach would prevent misunderstanding and strengthen the credibility of the methods section. Overall, your revisions move the manuscript in a positive direction, and further clarification on this point will help ensure complete methodological transparency.

You may consider adding the following clarification to avoid any misunderstanding regarding your screening methodology: “Our screening approach followed resource efficient methods as described by Garritty et al. (2022), which support single reviewer screening with partial independent verification. This method maintains high accuracy while reducing workload.”

This wording clearly indicates the methodological basis for your screening process and helps ensure that readers do not assume adherence to more traditional full double screening models.

Response: *Thank you for your valuable addition.* *We have revised the study selection paragraph in the manuscript to clarify the screening procedure according your suggestion.*

Revisions: Method section, page 5, line 97-98. The online software Covidence (www.covidence.org) [29] was used to support for the screening and selection process of papers according to international guidelines [28, 30]. **Screening was primarily performed by one reviewer with partial independent verification, an approach considered methodologically acceptable while enhancing feasibility [31].**

**Reviewer 2**

Comment: The authors have provided clear, thoughtful, and comprehensive responses to all reviewer comments. The revisions substantially improve the clarity, structure, and contextual depth of the manuscript. Overall, the responses are satisfactory, and the revisions meaningfully address the concerns raised. The manuscript is notably improved and is closer to being suitable for publication.

Response: Thank you for your positive feedback and for recognizing the substantive improvements made in response to the initial review comments.
